# Psychiatric referrals to the general hospital emergency department: are we being effective?

**DOI:** 10.3389/fpsyt.2023.1166191

**Published:** 2023-08-02

**Authors:** Shikma Keller, Einat Tilbor, Afnan Shwiki, Sharon Florentin, Sofia Laufer, Omer Bonne, Laura Canetti, Inbal Reuveni

**Affiliations:** ^1^Department of Psychiatry, Hadassah Medical Center and Faculty of Medicine, Hebrew University of Jerusalem, Jerusalem, Israel; ^2^Department of Psychology, Hebrew University of Jerusalem, Jerusalem, Israel

**Keywords:** general hospital emergency department, overcrowding, mental health, psychiatric emergencies, appropriate referrals, emergency psychiatry, general hospital psychiatry

## Abstract

**Introduction:**

General hospital emergency departments (GHEDs) are notoriously overcrowded. This is caused, in part, by ineffective referrals, that is to say referrals that do not require medical examination or other interventions in the context of a general hospital. This study aims to investigate the contribution of psychiatric referrals to this issue, to identify potential determinants of these referrals and offer means to reduce them.

**Materials and methods:**

Retrospective data were collected from psychiatric admission files within a GHED of a tertiary-care city hospital over a 1 year period. Two experienced clinicians separately reviewed each file to determine rationale of referrals according to predetermined criteria.

**Results:**

A total of 2,136 visits included a psychiatric examination, 900 (42.1%) were determined “effective,” and 1,227 (57.4%) were deemed “potentially ineffective.” The leading causes for potentially ineffective referrals to a GHED were psychiatric illness exacerbation (43.4%), and suicidal ideations (22%). Most referrals (66.9%) were initiated by the patient or their family, and not by a primary care physician or psychiatrist.

**Conclusion:**

More than half of the psychiatric referrals did not necessarily require the services of a general hospital, and may be more suitable for referral to a dedicated psychiatric facility. Ineffective referrals to the GHED pose a burden on general hospital resources, and may be less effective for the psychiatric patients. This calls for clear guidelines for the provision of optimal emergency treatment for mental-health patients.

## Introduction

Overcrowding of general hospital emergency department (GHED), in part caused by ineffective referrals, is a significant public health problem worldwide (1, 2). Overcrowding of GHEDs results in longer wait times and adverse health outcomes (3). Patients with psychiatric symptoms constitute a substantial proportion of GHED visits ranging from 3.4 to 12.5% of the overall workload of the GHED (4–7). These include patients with psychiatric illnesses, such as affective, anxiety or psychotic disorders, substance misuse, and/or trauma (7). Patients who are referred to a GHED are largely treated by medical staff who may be less proficient in managing patients experiencing a mental-health crisis compared to dedicated psychiatric facilities (8). The length of stay (LOS) for a psychiatric patient in the GHED is 3.2 h longer compared to non-psychiatric patients (9). Longer LOS has been found to be associated with more violent behavior among psychiatric patients, possibly due to the busy GHED environment which can exacerbate mental distress (8–10). Therefore, clarifying which patients need to be referred to a GHED, and which should receive care in dedicated psychiatric facilities, is crucial to reduce GHED workload and minimize harm to patients.

Current research reveals a prevalence of between 20 and 40% of inadequate referrals to emergency departments (EDs) (11, 12). However, these studies focus on medical, rather than behavioral circumstances. Nevertheless, causes of ineffective referrals may be applicable to psychiatry as well, including the use of GHED as a substitute for primary care, mainly due to shortage in primary care services or the need for services outside regular hours. Ensuring consistent primary health care has the potential to effectively address acute conditions and manage chronic disorders, diminishing the likelihood of exacerbations and need for urgent care (13). Further research into the specific needs of individuals with psychiatric illness is needed to ensure appropriate and timely care.

A psychiatric emergency is defined by the American Psychiatric Association as “an acute disturbance in thought, behavior, mood, or social relationship, which requires immediate intervention” (5), and refers first and foremost to the behavior of an individual and/or subjective feelings. Therefore, if an individual or his/her family seek help for a psychiatric emergency, it should always be available. Nevertheless, identifying patients that requires the services of a GHED to determine whether there is a physical illness that may be causing or exacerbating their psychiatric symptoms, as well as identify acute medical comorbidity that may occur together with psychiatric symptomatology, is crucial (14, 15). Consensus recommendations of the American Association for Emergency Psychiatry Task Force (15) advise new-onset psychiatric symptoms after the age of 45, patients 65 years and older, patients with delirium or cognitive deficits, focal neurological findings or evidence of head injury, substance intoxication, withdrawal, or exposure to toxins/drugs, decreased level of consciousness and abnormal vital signs, are at risk for a medical cause for their psychiatric symptoms, and therefore should undergo a physical evaluation in addition to a psychiatric examination (15). However, it is not known to what degree these recommendations are adhered to in clinical practice. Nevertheless, these recommendations suggest who may be these patients that will not require medical evaluation, and could undergo psychiatric assessments in a dedicated psychiatric service to decrease the ever-growing burden imposed upon the GHEDs.

In the current study we aimed first, to describe the current status of psychiatric referrals to a GHED of a tertiary-care hospital, and to determine how many of the referrals were effective, that is to say referrals that required medical evaluation and GHED facilities. Second, we aimed to identify psychosocial determinants associated with effective compared to potentially ineffective referrals to the GHED. The hypotheses were based upon clinical practice, as there are no previous studies addressing this issue. We hypothesized that the majority of cases referred to the GHED will not be in accordance with the above-mentioned recommendations, and may be appropriate for referral to dedicated psychiatric facilities, rendering them “potentially ineffective” referrals.

## Materials and methods

### Setting

The Hadassah Ein-Kerem Medical Center is a tertiary care hospital serving a catchment area population of 1 million inhabitants living in Jerusalem and its nearby area. Emergency psychiatric services in the GHED are available 24/7. Individuals who arrive at the ED are triaged at arrival according to their main complaint. Triage could be directly to a psychiatrist or any other physician from other disciplines who could later ask for psychiatric consultation, if needed. Laboratory or imaging tests are carried out, when necessary, to rule out or clarify medical causes for psychiatric symptoms. Israel’s health-care system provides every Israeli citizen health care service under the National Health Insurance Law. Emergency psychiatric services are provided in general hospitals EDs or in dedicated psychiatric emergency rooms located in regional psychiatric hospitals.

### Data

Retrospective anonymous data was collected from the admission files. Files of all the patients who were examined by a psychiatrist in the GHED in Hadassah Ein-Kerem Hospital over a 1 year period, between October 1st, 2015 and September 30th, 2016 were included. Filles were excluded from analysis due to major deficits in data (*n* = 9). Sociodemographic characteristics, past medical and psychiatric history, source of referral, presenting complaint, examination by a non-psychiatric physician, laboratory or imaging tests, diagnosis at discharge and visit outcomes were extracted and entered into an IBM SPSS Statistics database. Two experienced psychiatrists (SK and AS) separately assessed each referral, to determine whether it was an effective referral. When needed, a case-by-case discussion was conducted between raters until an agreement on the classification of the case was reached. The decisions were based primarily on the recommendations of the American Association for Emergency Psychiatry Task Force on Medical Clearance of Adult Psychiatric Patients (15), with additions which were based on clinical experience. The classification of a referral as “effective” was done according to the following criteria: (1) Suicide attempt, (2) Pregnant and postpartum women with behavioral complaints, (3) Patients presenting to the GHED following significant self-harm, (4) Suspected first psychotic episode, (5) Acute behavioral changes after the age of 60, (6) Psychiatric patients with comorbid physical illness, (7) Patients who arrived to the GHED after exposure to a traumatic event (e.g., terror attack), (8) Acute intoxication, and (9) Mixed behavioral and physical symptoms. All referral that did not meet the above listed criteria were defined as “potentially ineffective” referrals.

The study was reviewed and approved by the Hadassah Hebrew University Medical Center Ethics Committee. Written informed consent was not required in accordance with institutional and national policies.

### Statistical analyses

Descriptive analysis and examination of the distributional properties of socio-demographic and clinical variables were carried out. To compare appropriate versus inappropriate referrals, we used *t*-tests for independent samples with continuous variables and χ2 with categorical variables. In the case of more than two categories, χ2 was followed by *z*-tests for independent proportions with Bonferroni correction. Reported *p* values are two-sided. All analyses were performed using IBM SPSS 21.0 (IBM Corp 2012) statistical software. The level of statistical significance was set at 5% (two-sided) for all tests.

## Results

Out of a total of 71,706 referrals to the Hadassah Ein-Kerem GHED, 2,136 (2.98%) underwent a psychiatric examination. 900 (42.1%) of the referrals were defined as “effective,” according to the criteria defined above. 1,227 (57.4%) of the referrals were considered “potentially ineffective,” with an additional of 9 (0.4%) that we were excluded due to lack of information.

### Causes of referrals

**Figure 1:**
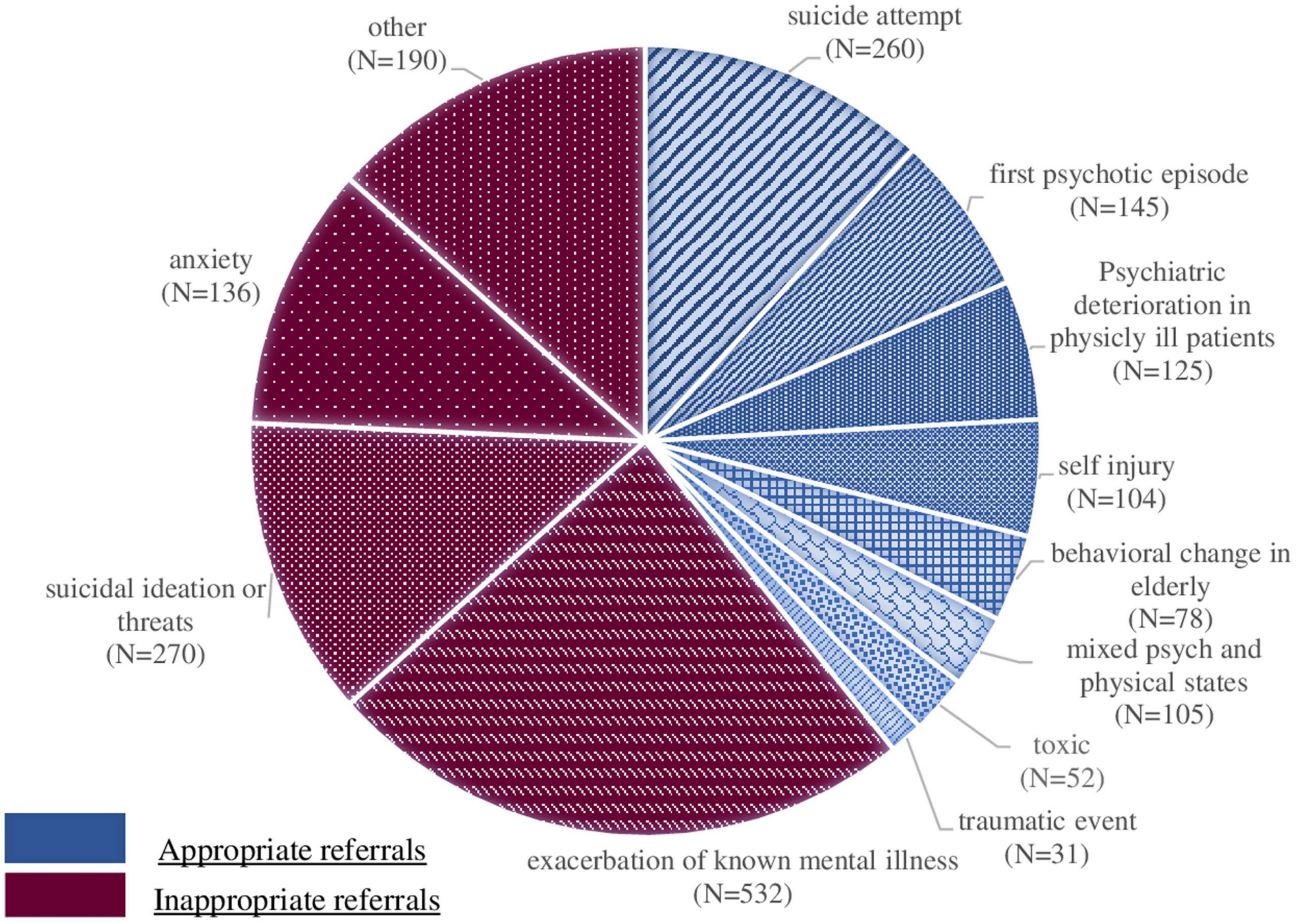
Effective and potentially ineffective causes of referrals to GHED.

Effective referrals to the GHED included individuals referred due to suicide attempts (28.9%), first psychotic episode (16.1%), psychiatric deterioration in physically-ill patients (13.9%), self-injury (11.1%) and behavioral change in the elderly (8.7%). The main causes of potentially ineffective referrals were: exacerbation of known mental illness (43.4%), specifically 17.6% due to psychotic exacerbation, 22% due to suicidal ideation or threats (without significant self-harm or suicide attempts), and 11% due to anxiety symptoms. See Figure 1 for more details on effective and ineffective causes for referral to the GHED. As shown in Table 1, medical history was significantly associated with more effective GHED referrals, while psychiatric history was related to more potentially ineffective referrals. Psychiatric diagnoses related to more potentially ineffective referrals were: schizophrenia, developmental/conduct disorders, and anxiety disorders. Whereas, personality disorders, dementia and mental disorders due to physical conditions, and referrals with no psychiatric diagnosis were associated with more effective referrals.

**Table 1:** Effective psychiatric referrals to GHED and previous medical, psychiatric history and diagnosis.

	Total *n* = 2,127	Effective referrals *n* = 900	Potentially ineffective referrals *n* = 1,227	*χ^2^*	*df*	*p*
Known medical history	769 (36.3%)	347 (38.8%)	422 (34.5%)	4.07	1	0.044
Previous medical hospitalization	583 (27.6%)	267 (29.9%)	316 (26.0%)	3.88	1	0.055
Known psychiatric history	1,487 (70.1%)	590 (65.8%)	897 (72.2%)	13.71	1	<0.001
Previous psychiatric hospitalization	783 (37.0%)	323 (36.2%)	460 (37.7%)	0.50	1	0.494
Previous psychiatric diagnosis				168.22	9	<0.001
None	548 (25.9%)	272 (30.4%)	276 (22.6%) *			
Schizophrenia	370 (17.5%)	92 (10.3%)	278 (22.8%) *			
Affective disorders	323 (15.3%)	123 (13.7%)	200 (16.4%)			
Personality disorders	318 (15.0%)	184 (20.5%)	134 (11.0%) *			
Developmental/Conduct disorders	189 (8.9%)	53 (5.9%)	136 (11.2%) *			
Anxiety and stressor related disorders	146 (6.9%)	53 (5.9%)	93 (7.6%) *			
Drug induced mental disorders	118 (5.6%)	68 (7.6%)	50 (4.1%) *			
Eating disorders	49 (2.4%)	15 (1.7%)	34 (2.8%)			
Dementia & mental disorders due to physical conditions	35 (1.7%)	33 (3.7%)	2 (0.2%) *			
Other mental disorder	19 (0.9%)	3 (0.3%)	16 (1.3%) *			

*Columns proportions significantly differ from each other.

### Psychosocial determinants associated with effective referrals

The socio-demographic characteristics and psychosocial correlates of the effective and ineffective referrals are shown in Table 2. Being male, Israeli-born, single, and unemployed were significantly associated with more potentially ineffective referrals. Being retired was significantly related to more effective ones. Referrals of patients who are 19 years old or younger, 30 to 39 years old, and 50 to 59 years old were more likely to be potentially ineffective, while referrals of patients aged 60 years or older were significantly associated with being more effective. Living in a rehabilitation facility was associated with more effective referrals.

**Table 2:** Psychosocial correlates with effectiveness of referral to GHED.

	Total *n* = 2,127	Effective referrals *n* = 900	Potentially ineffective referrals *n* = 1,227	*χ^2^/t*	*df*	*p*
Male	1,070 (50.3%)	413 (45.9%)	657 (53.6%)	12.31	1	0.001
Born in Israel	1,630 (77.1%)	652 (73.0%)	978 (80.0%)	14.39	1	<0.001
Single	1,235 (58.3%)	486 (54.5%)	749 (61.1%)	9.10	1	0.003
Employment status				48.28	2	<0.001
Employed	532 (26.0%)	231 (26.8%)	301 (25.4%)			
Unemployed	1,372 (67.0%)	533 (61.8%)	839 (70.8%) *			
Retired	144 (7.0%)	99 (11.5%)	45 (3.8%) *			
Age mean		38.51 ± 20.84	33.57 ± 16.18	5.93	2,125	<0.001
Age categories				74.38	5	<0.001
≤19	390 (18.3%)	139 (15.4%)	251 (20.5%) *			
20–29 y	603 (28.3%)	266 (29.6%)	337 (27.5%)			
30–39 y	431 (20.3%)	158 (17.6%)	273 (22.2%) *			
40–49 y	231 (10.9%)	90 (10.0%)	141 (11.5%)			
50–59 y	188 (8.8%)	65 (7.2%)	123 (10.0%) *			
≥60	284 (13.4%)	182 (20.2%)	102 (8.3%) *			
Living in a rehabilitation facility	194 (9.2%)	95 (10.6%)	99 (8.1%)	3.96	1	0.047

*Columns proportions significantly differ from each other.

### Characteristics of GHED referrals

Most of the referrals (66.9%) were initiated by the patients or their families, 13% by a non-psychiatric doctor, 5.98% by a psychiatrist, and 14.2% were brought to the ED by ambulance or police. Hours of visit at GHED: about a third, 717 (33.6%) approached the ED between 7:00 AM and 2:59 PM, the majority, 982 (46%) between 3:00 PM and 10:59 PM, and only 434 (20.4%) between 11:00 PM and 6:59 AM. For the majority of the sample (1,622, 76.1%) this was the first referral in 6 months, 256 (12%) had two referrals, and 253 (11.9%) had three or more referrals to GHED in the last 6 months. No significant associations were found between effective referrals and the source of referral (χ^2^_(3)_ = 1.64, *p* = 0.65), hours of visit (χ^2^_(2)_ = 2.31, *p* = 0.316), or frequency of referrals to the GHED (χ^2^_(2)_ = 4.40, *p* = 0.111).

## Discussion

As far as we know, this is the first study to investigate the effectiveness of referrals of adult psychiatric patients to a GHED, and to determine their nature, its magnitude and related characteristics. Our results demonstrate that about 58% of patients referred to the GHED during the study period did not need the facilities of a general hospital, and could have been referred directly to ED services in dedicated psychiatric facilities, and were therefore deemed as potentially ineffective. The leading causes for potentially ineffective referrals to the GHED were exacerbation of known mental illness (43.4%) and suicidal ideation (without significant self-harm behaviors and/or suicide attempts) (22%). A previous study of inappropriate pediatric psychiatric ED visits showed similar rates, defining only 39% of referrals as “fully appropriate.” Multivariate predictors of inappropriate referrals included children with suicidal ideation or attempts, low harm potential and severity of presenting complaint, and pediatric patients without diagnosis of psychosis (16). The high proportion of potentially ineffective referrals to the GHED could be explained by several factors. First, it may reflect a shortage of adequate and accessible dedicated psychiatric services in the community (17). Research shows that many psychiatric crises could have been managed successfully in a primary-care setting (13). Second, perceived stigma associated with being in psychiatric care or approaching a dedicated psychiatric facility could also increase patients’ preferences for a GHED (18–20). Increasing public awareness of mental health to reduce stigma may increase willingness to approach psychiatric services and psychiatric EDs. Finally, inadequate continuity of care and lack of communication between community-care providers and hospital staff could contribute to ineffective referrals to the GHED (21). Educating primary care physicians, individuals with psychiatric illnesses and their families regarding the indications for psychiatric hospitalization, and in which cases the services of a GHED may be needed to further evaluate medical causes for the psychiatric symptoms is warranted. Health-care organizations should have clear guidelines for physicians to help reduce ineffective self-referrals through policy and insurance payments.

About a third of referrals to the GHED in this study were due to a suicide attempt, suicidal ideations or self-harm thoughts or actions. Studies show that the point prevalence of active suicidal ideation can be up to 8% among GHED referrals presenting with non-psychiatric complaints. Suicide screening of all patients attending the GHED should be considered since it has the potential to improve identification and apply interventions to reduce subsequent suicidal behavior (22). Furthermore, it is estimated that up to 25% of United State (US) patients that visit GHEDs due to suicidal attempt, will make another attempt (23). In the ED-SAFE study (24) conducted across multiple centers, involving patients who visited ED due to recent suicide attempts, showed that mere screening did not establish any significant change but an intervention that was implemented showed a significant reduction in subsequent suicide attempts, with a 5% absolute decrease in the proportion of patients attempting suicide and a 30% decrease in the total number of suicide attempts over a 52 weeks follow-up period compared to treatment as usual (24). ED visits offer a window of opportunity to deliver prevention interventions, and to provide rapid referral to outpatient care. US national statistics indicate that only roughly half of youth presenting to GHEDs with suicidal ideation or self-harm, receive outpatient treatment after discharge. There is a need for programs that can be an alternative to the GHED for people who are at risk, for example mobile emergency psychiatric services and a mobile crisis intervention team. Furthermore, Individuals who approach the GHED after a suicide attempt should be offered extended observation for up to 72 h. This approach could provide time for evaluation, monitoring, and a brief intervention that could reduce future attempts (23) and thus will reduce GHED visits.

Our results show being single and unemployed was also associated with a higher likelihood of more potentially ineffective referrals. Previous studies of frequent visitors to psychiatric EDs suggest being single, living alone, and being economically disadvantaged may be related to a lack of social support and social isolation (25, 26). However, the latter studies did not examine the adequacy of GHED visits. We did not find a significant relationship between the frequency of GHED visits and potentially ineffective referrals. These psychosocial characteristics may be related to the initial psychiatric diagnosis rather than the circumstances of the referral to the GHED. Future research is needed regarding sub-groups of patients who may be more likely to have potentially ineffective referrals, which may contribute to GHED workload and crowding. The majority of potentially ineffective referrals to the GHED in the study included patients with exacerbation of known psychiatric disorders. Yet, most of the referrals (66.9%) were initiated by patients or their families, and not by their primary-care physician or psychiatrist. It would be expected that individuals with existing mental illness would be under psychiatric follow-up, and would be referred to the ED when the treating psychiatrist decides that the mental condition warrants hospitalization. This may attest to the shortage of outpatient psychiatric services in the community and therefore, expansion of psychiatric service in the community is needed. Self-referrals are one of the major causes of overcrowding of EDs and improper use of emergency services (27). Studies show a majority of medical self-referrals are also inappropriate (28). A literature review regarding patients’ motives for self-referrals to EDs concludes that the most common motives for self-referrals include health concerns and the patients’ expectation for further investigations of their complaints in the ED (29). This would not constitute an appropriate reason for a referral to the ED, medical or psychiatric, but rather should undergo further evaluation in community-setting. Interventions that educate patients and families regarding their illness have been shown to significantly reduce nonurgent ED visits (29). Increasing patients’ knowledge and awareness regarding recommended care in case of symptom exacerbation and suicidal thoughts could reduce potentially ineffective GHED referrals (30). According to our findings, living in a rehabilitation facility was associated with more effective referrals. Therefore, we suggest that developing more community rehabilitation services for individuals with serious mental illness may help reduce ER visits during momentary crises. This suggestion is supported by evidence that people with serious mental illness who receive rehabilitation services for more than one year show a decrease in the mean number of psychiatric hospitalization days per year (31). Health care policy should promote services that provide accessible alternatives to the GHED. Channeling patients to easily accessible, community services or urgent care centers, may also help mitigate GHED workload and allow more time and resources to treat populations with psychiatric symptoms, such as pregnant women, patients with eating disorders, young children, and geriatric patients, who should be evaluated in a GHED to exclude physical medical issues that require immediate attention (32, 33).

### Strengths and limitations

The strength of this study is in the comparably large sample size. As to the limitations, the study was based on hospital records. Therefore, the information presented here is limited. The effectiveness of the referrals was categorized by clinicians retrospectively and may be subjected to bias. Furthermore, the Israeli healthcare system provides universal healthcare coverage, including psychiatric services through public healthcare providers. Psychiatric emergency services are available in GHED and dedicated psychiatric facilities. This may limit generalizability to countries with different healthcare policies. Lastly, data presented in this study has been collected during 2015. Therefore, changes that may have occurred during this time, such as the pandemic and economic turmoil following it, may affect the results and need to be taken into account when interpreting the results.

## Conclusion

The number of potentially ineffective referrals of psychiatric patients to GHED is substantial, and represents a major public-health concern due to its substantial burden on GHED workload and negative effect on the psychiatric patient’s well-being and quality of care. Our results suggest that available recommendations for differential referral to psychiatric versus general hospital emergency care should be clearer. Guidelines for referrals of psychiatric patients in need by community service providers and patients alike are needed.

## Data availability statement

The raw data supporting the conclusions of this article will be made available by the authors, without undue reservation.

## Ethics statement

The study was reviewed and approved by the Hadassah Hebrew University Medical Center Ethics Committee. Written informed consent was not required in accordance with institutional and national policies.

## Author contributions

ET and AS collected the data from the admission files. SK and AS determined clinical effectiveness of all admission files. OB and SL contributed to the experimental design. IR, SK, and SF wrote the manuscript. LC done the statistical analysis. All authors contributed to the article and approved the submitted version.

## Funding

This research was funded by the Israel National Institute for Health Policy Research (grant number 2016/192).

## Conflict of interest

The authors declare that the research was conducted in the absence of any commercial or financial relationships that could be construed as a potential conflict of interest.

## Publisher’s note

All claims expressed in this article are solely those of the authors and do not necessarily represent those of their affiliated organizations, or those of the publisher, the editors and the reviewers. Any product that may be evaluated in this article, or claim that may be made by its manufacturer, is not guaranteed or endorsed by the publisher.
